# Development of quantification software for evaluating body composition contents and its clinical application in sarcopenic obesity

**DOI:** 10.1038/s41598-020-67461-0

**Published:** 2020-06-26

**Authors:** SeungJin Kim, Tae-Hoon Kim, Chang-Won Jeong, ChungSub Lee, SiHyeong Noh, Ji Eon Kim, Kwon-Ha Yoon

**Affiliations:** 1 Medical Convergence Research Center, Wonkwang University, Iksan, 54538 Republic of Korea; 2 Smart Health IT Center, Wonkwang University Hosptial, Iksan, 54538 Republic of Korea; 3 Department of Radiology, Wonkwang University School of Medicine, Wonkwang University Hosptial, Iksan, 54538 Republic of Korea

**Keywords:** Magnetic resonance imaging, Obesity, Software

## Abstract

In sarcopenic obesity, the importance of evaluating muscle and fat mass is unquestionable. There exist diverse quantification methods for assessing muscle and fat mass by imaging techniques; thus these methods must be standardized for clinical practice. This study developed a quantification software for the body composition imaging using abdominal magnetic resonance (MR) images and compared the difference between sarcopenic obesity and healthy controls for clinical application. Thirty patients with sarcopenic obesity and 30 healthy controls participated. The quantification software was developed based on an ImageJ multiplatform and the processing steps are as follows: execution, setting, confirmation, and extraction. The variation in the muscle area (MA), subcutaneous fat area (SA), and visceral fat area (VA) was analyzed with an independent two sample T-test. There were significant differences in SA (p < 0.001) and VA (p = 0.011), whereas there was no difference in MA (p = 0.421). Regarding the ratios, there were significant differences in MA/SA (p < 0.001), MA/VA (p = 0.002), and MA/(SA + VA) (p < 0.001). Overall, intraclass correlation coefficients were higher than 0.9, indicating excellent reliability. This study developed customized sarcopenia-software for assessing body composition using abdominal MR images. The clinical findings demonstrate that the quantitative body composition areas and ratios can assist in the differential diagnosis of sarcopenic obesity or sarcopenia.

## Introduction

The term ‘sarcopenic obesity’ has been proposed to identify obesity with low skeletal muscle function and mass^1^. The current definitions of sarcopenic obesity combine sarcopenia [which was registered in the International Classification of Diseases (ICD-10-CM) in 2016]^2^ as defined through variable criteria, with the presence of obesity either defined as body mass index (BMI) or adiposity levels^3–6^. To date, there has been growing interest in sarcopenic obesity or sarcopenia. Several studies have reported that sarcopenia is closely associated with obesity^7,8^, physical disorders, a decline in quality of life^9^, metabolic complications, disease incidence^10,11^, and in particular, the treatment effects of cancer patients^12,13^. These studies indicate that more patients within the obese population have a weakened musculoskeletal system or increased fat mass in all age groups, and the risks and prevalence of sarcopenia increased with diseases such as liver fibrosis, obesity and metabolic syndrome^7,8,11^. However, current studies regarding the quantitative assessment of sarcopenia and/or sarcopenic obesity remain insufficient as diagnostic criteria and measurement techniques for muscle mass (or muscle loss) have not yet been established. Furthermore, it is unclear whether the specific mechanism of sarcopenic pathological responses involved in the decrement of muscle mass is aging^14,15^. Therefore, there is an unmet need for the establishment of a standardized method to assess muscle and fat mass.

Recent studies regarding diagnostic methods for sarcopenia or sarcopenic obesity have focused on the use of methods for the quantitative evaluation of body composition. Body composition imaging typically refers to the quantification of body fat and muscle mass, with evaluation methods including anthropometry, bioelectrical impedance analysis (BIA), and medical imaging^16–18^. Among these, the medical imaging techniques are regarded as the gold-standard in order to assess whole-body and specific regional muscle and fat mass. Medical imaging methods have included dual-energy X-ray absorptiometry (DEXA), computed tomography (CT), and magnetic resonance imaging (MRI). MRI, specifically shows great promise for quantifying the soft tissues including muscle, fat, nerve and ligaments and no ionizing radiation for patients^19–21^. There are only a few whole-body MRI studies focusing on quantification of actual body composition^22,23^. However, the application of whole-body MRI is restricted in clinical settings as the manual assessment of whole-body organ and tissue mass is time consuming. Several studies have investigated the use of single slices to estimate whole-body composition as an alternative method. Quantified composition on individual slices obtained at the lumbar spine showed a strong correlation with total visceral fat, subcutaneous fat and muscle mass^24–27^. Adipose tissue areas 5–10 cm above L4–L5 showed the strongest correlation with total visceral fat volume, whereas there was no association with subcutaneous fat areas. The area ~ 5 cm above L4–L5 was established as a predictor of total body-muscle volume^28^. In both genders, a single MRI scan at the level of the third lumbar spine (L3) is the best compromise as a site to assess total volume of visceral fat, subcutaneous fat and muscle^24^. Therefore, the use of single slice and automatic quantification software with a rapid processing time is useful for clinical implementation. Currently, there are few studies focusing on the quantification of body composition in sarcopenic obesity or sarcopenia.

For this study, we developed a semi-automatic quantification software for body composition imaging using abdominal MR images and compared the differences between sarcopenic obesity and healthy controls for clinical application.

## Results

### Patient characteristics

The averaged enzyme levels in both groups are shown in Table 1. The serum biochemistry showed significant differences between the two groups as follows: aspartate aminotransferase (AST, *p* < 0.001), alanine aminotransferase (ALT, *p* < 0.001), fasting glucose (*p* = 0.014) and triglycerides (TG, *p* < 0.001). However, there was no significant difference in γ-glutamyl transpeptidase (GGT, *p* = 0.249) or alkaline phosphatase (ALP, *p* = 0.170). Compared with those of healthy controls, these changes in serum levels can closely suggest the changes in the metabolic status such as those in sarcopenia and obesity.

**Table 1:** General characteristics in sarcopenic obesity and healthy control groups.

	**Sarcopenic obesity (N = 30)**	**Healthy control (N = 30)**	p-value*
Demographical characteristics (mean ± SD)			
Age (years)	47 ± 19	55 ± 17	0.132
BMI (body mass index, kg/m^2^)	29.4 ± 2.5	21.5 ± 1.6	< 0.001
Height (m)	1.6 ± 0.1	1.6 ± 0.1	0.025
Weight (Kg)	75.8 ± 12.8	59.5 ± 7.7	< 0.001
Blood chemistry (mean ± SD)			
Aspartate aminotransferase (AST, IU/L)	72.8 ± 38.7	33.3 ± 16.0	< 0.001
Alanine aminotransferase (ALT, IU/L)	121.8 ± 90.4	34.7 ± 42.6	< 0.001
γ-glutamyl transpeptidase (GGT, IU/L)	72.4 ± 50.7	112.8 ± 170.0	0.249
Fasting glucose (mg/dL)	118.3 ± 31.2	89.8 ± 20.4	0.014
Triglyceride (TG, mg/dL)	211.4 ± 118.6	102.5 ± 35.0	< 0.001
Alkaline phosphatase (ALP, IU/L)	284.5 ± 84.6	400.0 ± 362.5	0.170

*The difference between normal control and sarcopenia disease groups was analyzed by the independent two sample T-test.

### Measurements of body composition contents using developed software

**Figure 1:**
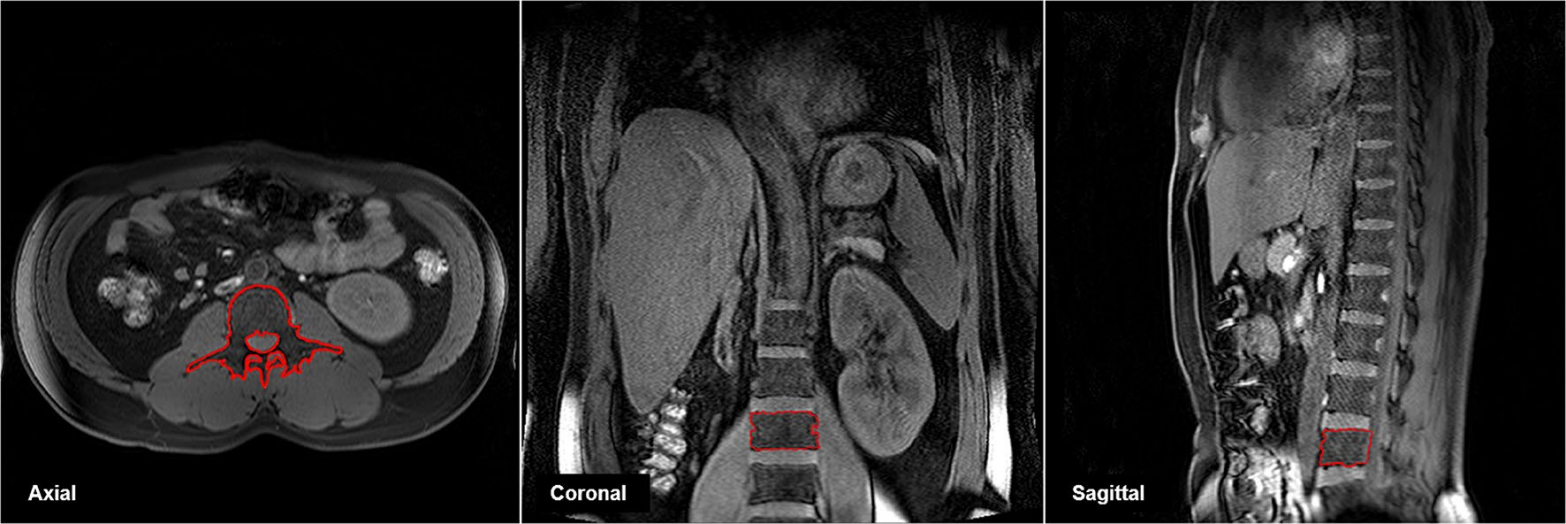
Abdominal MR images at third lumbar spine (L3, inside red-line) level.

**Figure 2:**
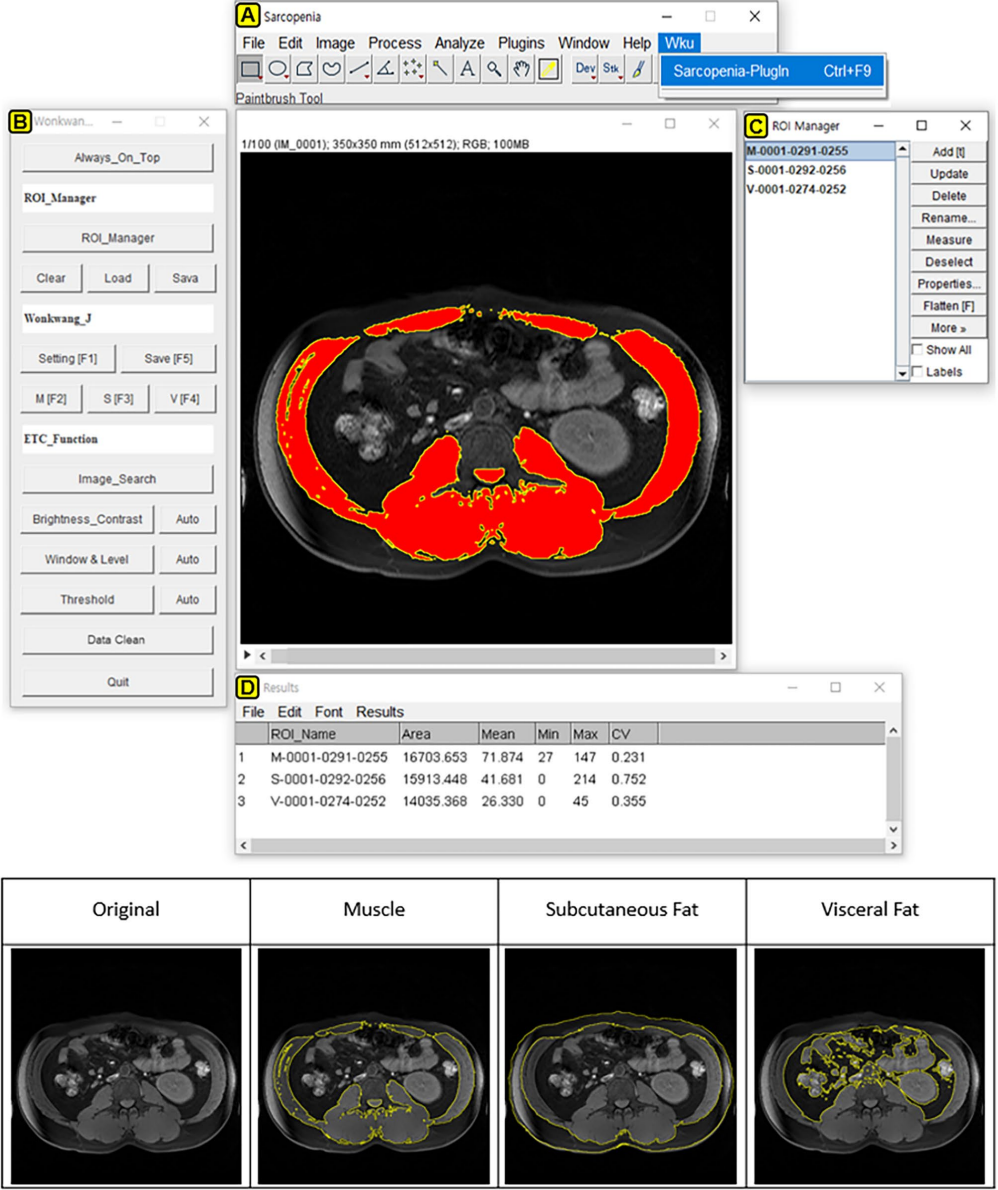
The graphic user interface (GUI) of developed software ‘sarcopenia plug-in’ (upper) including of (**A**) ImageJ-platform basic menubar, (**B**) sarcopenia plug-in window, (**C**) region of interest (ROI) manager window and (**D**) result window. An example (lower) demonstrated the ROI extraction (inside yellow-line) for quantifying muscle, subcutaneous fat and visceral fat mass in a patient with sarcopenic obesity using developed ‘sarcopenia plug-in.’ The original ImageJ software (ver.1.51t, Java 1.8.0_191 64bits) is available at https://imagej.nih.gov/ij/.

**Figure 3:**
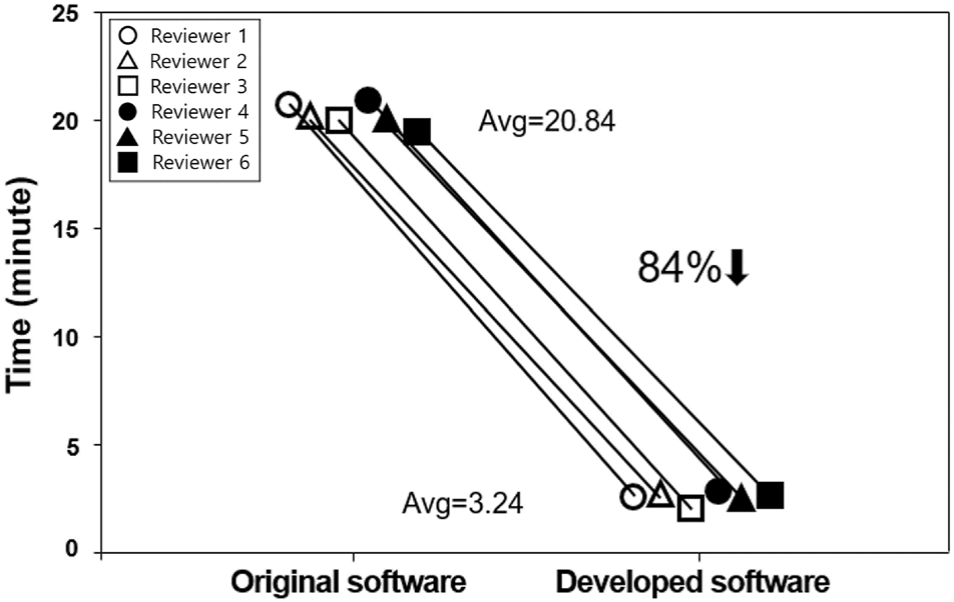
Comparison of total processing time obtained from 6 independent reviewers using original software (Avg. 20.84 min) and developed software (Avg. 3.24 min).

Five anonymized MRIs with the same slice at the L3 location (Fig. 1) were selected and provided to six reviewers. Each reviewer independently analyzed the major composition contents in sarcopenic obesity (i.e., muscle, subcutaneous fat, and visceral fat). Table 2 lists the processes for qualitative and quantitative analyses. Figure 2 demonstrates the graphic user interface (GUI) of the developed software and an example image for major composition contents. The mean time for quantifying muscle and fat areas was 20.84 min per image (range 19.56–21.84 min) for the original software and 3.24 min per image (range 2.86–3.36 min) for the developed software. The total processing time using the developed software was reduced by 6.43 times compared with the original software (84% reduced time: Fig. 3).

**Table 2:** Simplification in quantitative analysis processes on the developed software ‘sarcopenia plug-in’.

	**Original software**		**Developed software**
Execution	Sarcopenia Dicom Image Open	→	Sarcopenia Dicom Image Open (Auto Sarcopenia plugin playing)
Setting	Medical Image bit conversion	→	Medical Image condition value Setting and Conversion (bit, Window & Leveling, Threshold)
	Medical Image Window & Leveling value Setting		
	Medical Image Threshold value Setting		
	Setting ROI CreateSelection		Semi-Automatic Segmentation for Muscle and Fat Boundary
	Manual Segmentation for Muscle and Fat Boundary		
Confirm	Muscle and Fat ROI Extraction Calculation Process	→	Semi-Automatic Muscle and Fat ROI Extraction and Confirm
	Muscle and Fat ROI Confirm		
Extraction	Muscle and Fat ROI Extraction	→	Sarcopenia ROI, Area Quantification and Labeling Image Extraction
	Quantified Muscle and Fat Area Extraction		
	Muscle and Fat Labeling Image Extraction

### Differentiation of muscle and fat mass in sarcopenic obesity

**Figure 4:**
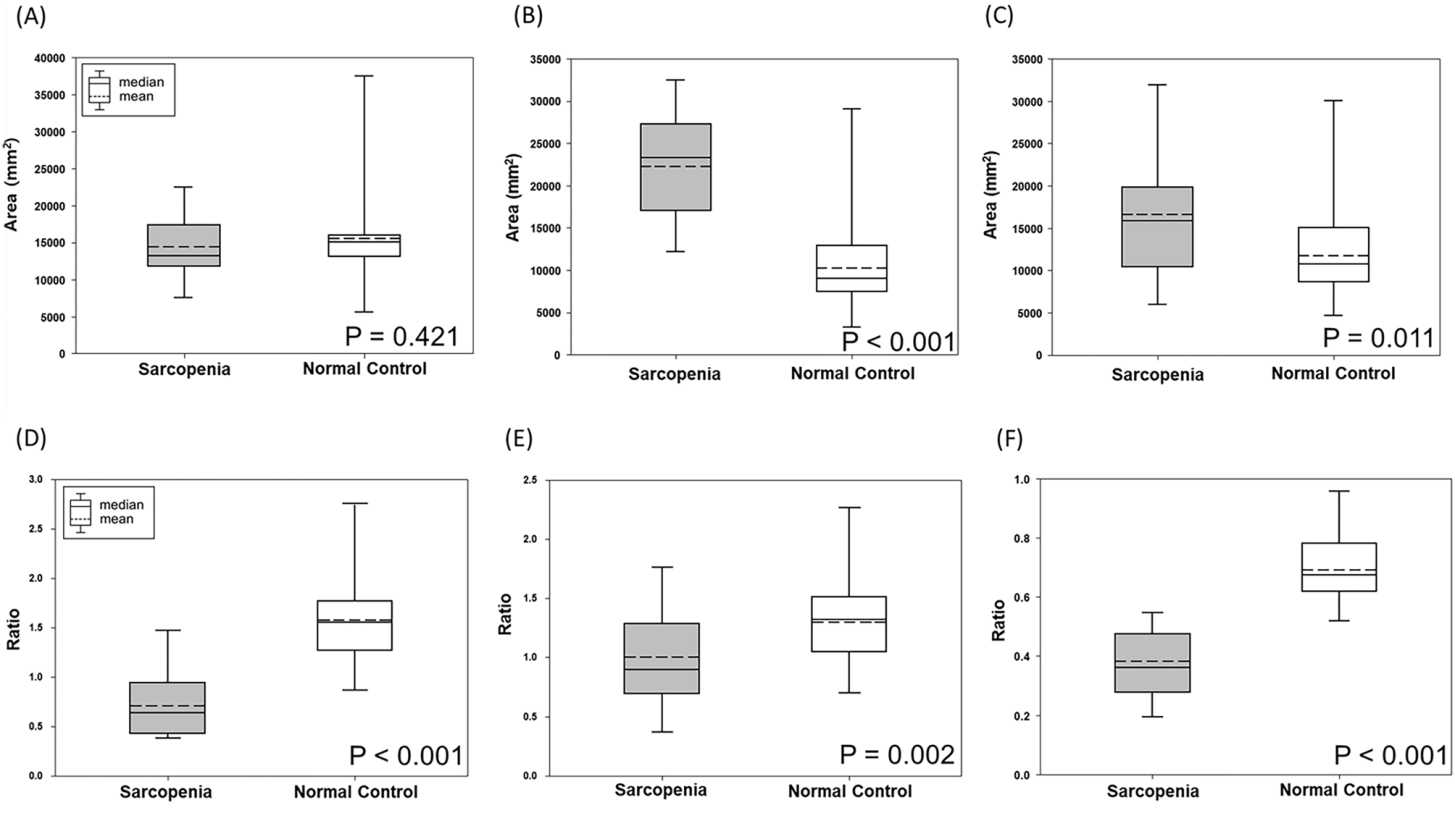
Box plots demonstrated the areas of muscle (MA, **A**), subcutaneous fat (SA, **B**), visceral fat (VA, **C**) and the ratios as MA/SA (**D**), MA/VA (**E**), MA/(SA + VA) (**F**) in sarcopenic obesity and healthy control groups using developed software ‘sarcopenia plug-in’.

MR image data in 30 patients with sarcopenic obesity and 30 healthy controls were analyzed with the developed software. The averaged areas (A) and ratio of muscle (M), subcutaneous fat (S), and visceral fat (V) in two groups are summarized in Table 3. There were significant differences in the subcutaneous fat area (SA); (*p* < 0.001; Fig. 4B) and the visceral fat area (VA); (*p* = 0.011; Fig. 4C), whereas no significant difference was found in the muscle area (MA); (*p* = 0.421; Fig. 4A). In the ratios, there were significant differences in MA/SA (*p* < 0.001; Fig. 4D), MA/VA (*p* = 0.002; Fig. 4E), and MA/(SA + VA); (*p* < 0.001; Fig. 4F). Therefore, the ratios derived from muscle and fat areas are expected to be more powerful indexes for distinguishing sarcopenic obesity to healthy control.

**Table 3:** Muscle and fat areas in sarcopenic obesity and healthy control groups.

	**Sarcopenic obesity (N = 30)**	**Healthy control (N = 30)**	p-value*
Muscle area (MA, mm^2^)	14,384.9 ± 3,684.5	15,492.2 ± 6,479.0	0.421
Subcutaneous fat area (SA, mm^2^)	22,337.2 ± 5,588.8	10,489.7 ± 5,222.7	< 0.001
Visceral fat area (VA, mm^2^)	16,224.6 ± 6,439.1	12,211.2 ± 5,132.4	0.011
MA/SA ratio	0.7 ± 0.3	1.6 ± 0.4	< 0.001
MA/VA ratio	1.0 ± 0.4	1.3 ± 0.3	0.002
MA/(SA + VA) ratio	0.4 ± 0.1	0.7 ± 0.1	< 0.001

Data present as mean ± standard devieation.

*The difference between normal control and sarcopenia disease groups was analyzed by the independent two sample T-test.

### Inter-rater agreement

The interobserver variability in muscle and fat areas between 2 observers is summarized in Table 4. There was no significant difference between the averaged muscle and fat areas of the 2 observers. Overall, intraclass correlation coefficients (ICCs) were higher than 0.9, indicating an excellent reliability. The ICCs (range 0.952–0.994) were 0.977 for the measurements in sarcopenic obesity patients and 0.971 for the healthy control group, respectively. Therefore, the overall muscle and fat measurements of both observers showed an excellent agreement (*p* < 0.001).

**Table 4:** Inter-rater variability in muscle and fat measurements.

	**Observer A**	**Observer B**	p-value*	**Intra-rater**	**95%CI**		**p-value†**
				**reliability (ICC)†**	**Lower bound**	**Upper bound**	
Overall (n = 60)							
MA	14,938.6 ± 5,277.1	15,214.5 ± 5,493.9	0.780	0.960	0.933	0.976	< 0.001
SA	16,413.4 ± 8,060.7	17,023.8 ± 8,343.1	0.684	0.994	0.989	0.996	< 0.001
VA	14,217.9 ± 6,174.5	14,694.2 ± 6,457.8	0.680	0.989	0.981	0.993	< 0.001
Sarcopenic obesity (n = 30)							
MA	14,384.9 ± 3,747.5	14,392.2 ± 3,786.1	0.994	0.985	0.968	0.993	< 0.001
SA	22,337.3 ± 5,684.4	22,907.1 ± 5,581.7	0.697	0.991	0.980	0.996	< 0.001
VA	16,224.6 ± 6,549.2	16,806.5 ± 7,089.6	0.742	0.992	0.982	0.996	< 0.001
Healthy control (n = 30)							
MA	15,492.2 ± 6,479.0	16,036.8 ± 6,758.3	0.751	0.952	0.899	0.977	< 0.001
SA	10,489.7 ± 5,222.7	11,140.5 ± 6,232.6	0.663	0.983	0.963	0.992	< 0.001
VA	12,211.2 ± 5,132.4	12,581.9 ± 5,034.9	0.779	0.979	0.957	0.990	< 0.001

*MA* muscle area, *SA* subcutaneous fat area, *VA* visceral fat area, *ICC* intraclass correlation coefficient, *CI* confident interval.

Areas quantified from each observer are presented as means ± SD.

*The differences between two observers in area measurements were assessed by the independent two sample T-test.

^†^The intra-rater reliability between two observers was assessed by the intraclass correlation (ICC) test.

## Discussion

This study developed semi-automated software for assessing body composition on the basis of ImageJ. The study analyzed the difference between people with sarcopenic obesity and a healthy control group by measuring body composition areas from retrospective MRI datasets. In our study, abdominal MR images with 3-dimensional T1 high-resolution isotropic volume excitation (THRIVE) pulse sequence demonstrated good discrimination in diagnosing sarcopenic obesity patients (as SA and VA; *p* < 0.05). Moreover, the ratios of muscle and fat areas with better discrimination are MA/SA, MA/VA and MA/(SA + VA) (*p* ≤ 0.002) compared to areas. Therefore, our findings demonstrate that the areas & ratios of muscle and fat composition quantified at the single slice level of L3 can be useful for diagnosing sarcopenic obesity.

This study investigated the reproducibility of inter-observer assessment. The muscle and fat areas measured by two observers were excellent with regard to inter-rater agreement (> 0.9), indicating reproducibility. Therefore, the sarcopenia software-based body composition measurements can be reproduced in clinical abdominal MR images. Compared with the original ImageJ program, the processing steps of developed software for quantification were simplified from 11 to 5 processes. Consequently, total processing times were greatly reduced to approximately 3 min per subject as shown in Fig. 2. Thus, our semi-automatic sarcopenia software with a rapid processing time would be beneficial for clinical implementation. Also, the advantage of the ImageJ program is that it is an open source platform based on the Java programming language. It provides high scalability through a Java plug-in and macro functionality. Further study is required, to test our software and other segmentation software such as the medical imaging interaction toolkit (MITK) and the imaging interaction toolkit (ITK). Further study could validate the performance efficacy of our software to measure voluminal muscle and fat in a similar manner and time.

With regard to our study design, this retrospective study used inclusive criteria on the basis of BMI^29^ and blood biochemistry from electrical health records (EHR) for enrollment of sarcopenic obesity patients. This retrospective enrollment may be considered a potential bias or variation. The potential risk factors in the selection of the patients (age, sarcopenic severity, gender and so on), the clinical conditions (initial management, drug type or dosage, treatment and etc.) and imaging setting (type of scanners/pulse sequences) or any combinations with the sources may represent bias. In the present study, the evaluation method for overweight obesity included the Korean standard BMI (> 25.0 kg/m^2^) in conjunction with elevated serum enzyme alanine aminotransferase (ALT) levels. The BMIs and ALT levels in the sarcopenic obesity group were higher than those in the healthy control group. Image-based fat quantification is well reflected in the differences in the subcutaneous and visceral fat in the patient group, but not in the MA. A recent study reported that T2 (or T2*-corrected) Dixon MRI and MR spectroscopy (MRS) techniques can provide reliable quantification of fat composition using proton density fat fraction (PDFF) and proton density water fraction (PDWF) while minimizing MR-specific effects^30^. Thus, this finding may be considered a good indicator for assessing the severity of obesity. Further cross-validation studies are essential in order to confirm the muscle/fat composition of a large cohort population together with other imaging methods.

This study had several limitations. First, our study dealt with middle aged subjects in both groups. Several studies reported that aging affects muscle mass and maximum muscular strength including individual differences^10,11^. In addition to the muscle/fat assessment, the volumetric muscle measurement and muscle composition must be quantitatively investigated as these factors can differ greatly between individuals. This depends on many confounding factors such as physical activity, smoking and nutrition^31,32^. However, in this study, there was no consideration for aging, physical activity, smoking and nutrition as potential variables, which could influence the evaluation of the muscle mass and muscular strength. Further correlative studies are essential in order to clarify the physiological responses and how those potential variables affect sarcopenic obesity. Moreover, we suggest a standardized study protocol/design with prospective, large-scale cohort investigations according to age group (20–60 years and above). Second, BMI assessment is the simplest method for assessing obesity. However, it is limited to evaluate actual body composition because the values are indirect indices based on body weight, height, and waist circumference (i.e., not the actual muscle and fat mass). The BMI cut-offs as criteria of overweight and obesity are different from those in the World Health Organization (WHO) Expert committee (25.0–29.9 kg/m^2^), the Western Pacific Regional Office (WPRO) of WHO (> 23.0 kg/m^2^), the Asia Pacific region (> 25.0 kg/m^2^) and other various countries^16^. Thus, a standardized index is required in order to accurately measure the actual amount of muscle and fat mass in patients with obesity or sarcopenia. To overcome this issue, we believe that our imaging-based quantification software could be a solution and could provide accurate muscle and fat information to physicians. Third, there is the issue of selecting a single slice at the L3 level for body composition measurement. Selecting a single slice instead of a whole-body MRI analysis may hold true in a cross-sectional study design. However, weight change estimates with a single slice cannot replace whole body assessments^24,33^. Thus, a single slice MR analysis should be carefully used to assess weight change in patients with sarcopenia. Also, MRI estimates of skeletal muscle mass are on mass rather than on tissue composition. The current issue of fat infiltration into muscle should be focused on actual muscle mass measurement. When compared with MRI, CT estimates include attenuation which provides further information of clinical relevance^34^. A solution could be a multivoxel MRS protocol for fat infiltration^35^. Fourth, most of the issues regarding sarcopenia relate to the skeletal muscles of the arms and legs (i.e., peripheral skeletal mass). This is again associated with the sequelae of sarcopenia, e.g., frailty. Thus, future studies must clarify the association between skeletal muscle estimates at L3 and the thigh.

In conclusion, this study developed a customized sarcopenia-software for assessment of body composition using abdominal MRI images. Our software has the advantages of use in an open source platform and a rapid quantification time for clinical application. The clinical findings demonstrate that the quantitative body composition data such as areas and ratios can assist in the differential diagnosis of obesity and in determining the ratios of muscle and fat. These could be considered as imaging biomarkers for sarcopenic obesity in clinical practice.

## Subjects and method

### Ethics statement

We conducted a retrospective study, which was approved by the institutional review board (IRB) of Wonkwang University Hospital. Written informed consent was exempted by the approval of Wonkwang University Hospital IRB committee due to the use of anonymous archival data including MRI data and the use of electronic health records for the application of the developed software. This study was conducted in accordance with the Helsinki Declaration and Good Clinical Practice.

### Study population

In compliance with the legal guidelines on safety and IRB bioethics, a total of 60 subjects consisting of 30 obese patients with suspected sarcopenia (mean age 47.3 ± 19.4 years.) and 30 healthy controls (mean age 54.6 ± 17.2 years.) were enrolled in this study from January 2014 to April 2019^36^. The individuals complained of fatigue and inactivity, and they appeared to be weaker than their maximum muscular strength. The Korean standard BMI (kg/m^2^) was used as the selection criteria^29^. Subjects were defined with the following BMI values in conjunction with serum enzyme levels^37^: suspected sarcopenic obesity group with an elevated serum ALT levels of (≥ 35 IU/L) and a BMI of at least 25.0 kg/m^2^; and a healthy control group with BMIs of 18.0–23.0 kg/m^2^ and a normal ALT level of (< 35 IU/L).

### Magnetic resonance imaging

Abdominal MR images were acquired from a 3 T Achieva MRI system (Philips Healthcare, Best, The Netherlands) with an array coil with 32 receiver channels. The T1 high-resolution isotropic volume excitation (eTHRIVE) images were obtained with the following parameters: repetition time (TR)/echo time (TE) = 4.2/1.97 ms; field of view = 38 × 38 × 14 cm^3^; number of excitation = 2; slice thickness = 0.74 × 0.74 × 2.0 mm^3^; number of slices = 100; matrix size = 512 × 512 pixels; and scan time = 16 s.

### Measurement of body compositions on 3rd lumbar spine MRI

To measure body compositions, this study chose a single slice analysis instead of a whole body MRI analysis with a cross sectional study design^24,33^. The L3 level image was selected as the position for quantitative analysis (Fig. 1). Not only are visceral and subcutaneous fat visible in this position, but also the seven major muscles (the psoas, erector spinae, quadratus lumborum, transversus abdominus, external and internal obliques, and rectus abdominus) can be identified. Moreover, this L3 level includes the spine, intestines, kidneys, and liver. Hence, it is a key position for observing various anatomical areas^24,28^, it is considered the most suitable position at which to analyze the relationship between various conditions and diseases including sarcopenia, aging, obesity, and osteoporosis^38^.

### Software environment and software algorithm

In order to quantitatively analyze the muscle and fat mass in the patients with sarcopenic obesity, the software was developed on the open source ImageJ multiplatform software (ver.1.51t, Java 1.8.0_191 64bits), developed by the National Institutes of Health (NIH)^39^. To use the software, the Java standard edition (SE) Runtime Environment is required to be installed in advance. Table 2 lists the overall processes for qualitative and quantitative analyses on the original ImageJ software and the developed sarcopenia-specialized software. The main processes are divided into four steps (execution, setting, confirmation and extraction), and the existing 11 analysis processes in the original software are simplified into five processes in the developed software.

### Data processing and quantification of MR images

The procedures for MRI data processing are comprised of four steps as follows: execution, setting, confirmation and extraction.

1. The execution step.
  MRI data are opened from the developed sarcopenia-software in this study and analysis tools specific to the quantification of body composition are implemented as shown in Fig. 2A. A L3 level image was chosen from the axial MR images in each patient to identify the ROIs of muscle, subcutaneous fat, and visceral fat.
2. The setting step.
  For the pre-processing of the MRI data, the selected MR image is set in the Window. The Leveling and Threshold values as shown in Fig. 2B (F1 function key button), and their values are applied to the MRI data. After the setting, the ROIs (as shown in Fig. 2C) corresponding to the muscle, subcutaneous fat and visceral fat are roughly drawn on the MR image using the drawing tools on menu bar (Fig. 2A).
3. The confirmation step.
  This is the step to confirm the final ROIs for each composition content (M, S, V) after modifying and verifying the ROIs. The final ROIs are generated from the overlaid areas between the roughly drawn ROIs and the regions within Threshold value. The function key buttons for generating the ROIs are as follows: F2 button for muscle (M), F3 button for subcutaneous fat (S) and F4 button for visceral fat (V), as shown in Fig. 2B.
4. The extraction step
  After the quantification of the body composition is completed for the confirmed ROIs from a physician (K.H.Y), the results are extracted into several file formats such as TIFF, PNG, JPG and BMP files. These files are used for the color-labeling of the ROI images, the ROI files for the confirmed ROIs, and the CSV files for the quantification data.

To compare the original software and developed software, five anonymized MRI images at the same slice of L3 location were selected and provided to six reviewers. Each reviewer independently analyzed the major composition contents in sarcopenia (i.e., muscle, subcutaneous fat, and visceral fat). They had no knowledge of the clinical outcome or access to the readings of the other reviewers. To assess the inter-observer variability of the measurements, both radiologists independently assessed the L3 images. The overall measurements for each patient were calculated as a mean and standard deviation of the areas.

### Statistical analysis

The abdominal muscle and fat contents were compared with two independent groups using the statistical package for the social sciences program (SPSS ver. 20, Chicago, Illinois). The variation in muscle and fat contents was evaluated with an independent two sample T-test. Inter-rater agreement and reliability were estimated by calculating the intra-class correlation coefficient (ICC); (and a 95% confidence interval [CI]) between the muscle and fat areas for the same subject on the same system. The ICC values were considered as the basis to evaluate the level of reliability using the following guideline^40^: values less than 0.5 are indicative of poor reliability, values between 0.5 and 0.75 indicate moderate reliability, values between 0.75 and 0.9 indicate good reliability, and values greater than 0.90 indicate excellent reliability. Two-sided *p* values less than 0.05 were considered to indicate statistical significance in all tests.
